# IKKβ Regulates the Repair of DNA Double-Strand Breaks Induced by Ionizing Radiation in MCF-7 Breast Cancer Cells

**DOI:** 10.1371/journal.pone.0018447

**Published:** 2011-04-07

**Authors:** Lixian Wu, Lijian Shao, Ningfei An, Junru Wang, Senthil Pazhanisamy, Wei Feng, Martin Hauer-Jensen, Shigeki Miyamoto, Daohong Zhou

**Affiliations:** 1 Division of Radiation Health, Department of Pharmaceutical Sciences, University of Arkansas for Medical Sciences, Little Rock, Arkansas, United States of America; 2 Department of Pharmacology, Fujian Medical University, Fuzhou, China; 3 Department of Pathology, Medical University of South Carolina, Charleston, South Carolina, United States of America; 4 Central Arkansas Veterans Healthcare System, Little Rock, Arkansas, United States of America; 5 Department of Pharmacology, University of Wisconsin-Madison, Madison, Wisconsin, United States of America; University of Medicine and Dentistry of New Jersey, United States of America

## Abstract

Activation of the IKK-NFκB pathway increases the resistance of cancer cells to ionizing radiation (IR). This effect has been largely attributed to the induction of anti-apoptotic proteins by NFκB. Since efficient repair of DNA double strand breaks (DSBs) is required for the clonogenic survival of irradiated cells, we investigated if activation of the IKK-NFκB pathway also regulates DSB repair to promote cell survival after IR. We found that inhibition of the IKK-NFκB pathway with a specific IKKβ inhibitor significantly reduced the repair of IR-induced DSBs in MCF-7 cells. The repair of DSBs was also significantly inhibited by silencing IKKβ expression with IKKβ shRNA. However, down-regulation of IKKα expression with IKKα shRNA had no significant effect on the repair of IR-induced DSBs. Similar findings were also observed in IKKα and/or IKKβ knockout mouse embryonic fibroblasts (MEFs). More importantly, inhibition of IKKβ with an inhibitor or down-regulation of IKKβ with IKKβ shRNA sensitized MCF-7 cells to IR-induced clonogenic cell death. DSB repair function and resistance to IR were completely restored by IKKβ reconstitution in IKKβ-knockdown MCF-7 cells. These findings demonstrate that IKKβ can regulate the repair of DSBs, a previously undescribed and important IKKβ kinase function; and inhibition of DSB repair may contribute to cance cell radiosensitization induced by IKKβ inhibition. As such, specific inhibition of IKKβ may represents a more effective approach to sensitize cancer cells to radiotherapy.

## Introduction

The IκB kinase (IKK)-nuclear factor κB (NFκB) pathway is one of the most important cellular signal transduction pathways [1]. It consists of members of the NFκB family and the family of inhibitors of NFκB (IκB), the IκB kinase (IKK) complex, and various other regulatory components. The NFκB family includes RelA (p65), RelB, c-Rel, NFκB1/p105 (p50 precursor), and NFκB2/p100 (p52 precursor); the IκB family consists of IκBα, IκBβ, IκBε, Bcl-3, p100/IκBδ, and p105/IκBγ; and the IKK complex is composed of two catalytic subunits, IKKα and IKKβ, and the regulatory subunit IKKγ. Normally, members of the NFκB family form a heterodimer/homodimer that resides in the cytoplasm as an inactive complex in association with a member of the IκB family. Upon stimulation with an inflammatory stimulus, the so-called canonical or classical pathway is activated, leading to the activation of IKK complex. Activated IKKα and/or IKKβ phosphorylate IκBα at S-32 and S-36. This causes IκBα ubiquitination and degradation by the S26 proteasome, thereby, allowing NFκB to translocate into the nucleus to regulate NFκB target genes. Through regulation of its target genes, NFκB can regulate various physiologic processes such as cell proliferation, migration and survival.

In addition, an increasing body of evidence suggests that activation of the IKK-NFκB pathway also play a pivotal role in the development of cancer resistance to ionizing radiation (IR) and chemotherapy [2]–[5]. This is because IR and many chemotherapeutic agents can activate NFκB through the atypical NFκB activation pathway by induction of DNA double-strand breaks (DSBs) [6], [7]. DSBs can activate ataxia telangiectasia mutated (ATM) that in turn phosphorylates IKKγ at Ser85. This leads to IKKγ mono-ubiquitination and translocation into the cytoplasm, where IKKγ remains associated with ATM to activate IKKα and/or IKKβ. It has been shown that activation of the IKK-NFκB pathway renders many types of tumor cells more resistant to IR and chemotherapy presumably via induction of anti-apoptotic proteins [2]–[5]. Therefore, inhibition of the NFκB transcriptional activity has been extensively exploited as a novel approach to sensitize cancers to radiotherapy and chemotherapy, but has achieved mixed results [2]–[5]. Therefore, further studies are urgently needed to gain a better understanding on how activation of the IKK-NFκB pathway regulates tumor cell sensitivity to IR and chemotherapy before a molecular targeted therapy against the IKK-NFκB pathway can be effectively employed for cancer treatment.

It has been well established that IR kills cancer cells primarily by induction of DSBs and efficient repair of DSBs is required for the clonogenic survival of irradiated cells [8], [9]. Therefore, we hypothesized that activation of the IKK-NFκB pathway by IR may also promote cancer cell survival in part by regulating the repair of DSBs. To test this hypothesis, we first used BMS-345541 (BMS), a specific IKKβ inhibitor [10], to selectively inhibit the IKK-NFκB pathway and found that it could significantly inhibit the repair of IR-induced DSBs in MCF-7 human breast cancer cells and H1299 and H1648 human lung cancer cells. Interestingly, the repair of IR-induced DSBs in MCF-7 cells was not affected by down-regulation of IKKα, but was significantly inhibited by IKKβ knockdown. In addition, the suppression of DSB repair by knockdown or inhibition of IKKβ was associated with an increased sensitivity of MCF-7 cells to IR. DSB repair function and resistance to IR were completely restored in IKKβ-knockdown MCF-7 cells after reconstitution with an active form of IKKβ. To our knowledge, this is the first study demonstrating that activation of the IKK-NFκB pathway by IR can regulate the repair of DSBs and inhibition of IKKβ activity may sensitize cancer cells to IR at least in part via inhibition of DSB repair. Therefore, specific inhibition of IKKβ represents a more effective approach to sensitize cancer cells to radiotherapy.

## Results

### IKKβ inhibition suppresses the repair of IR-induced DSBs

**Figure 1:**
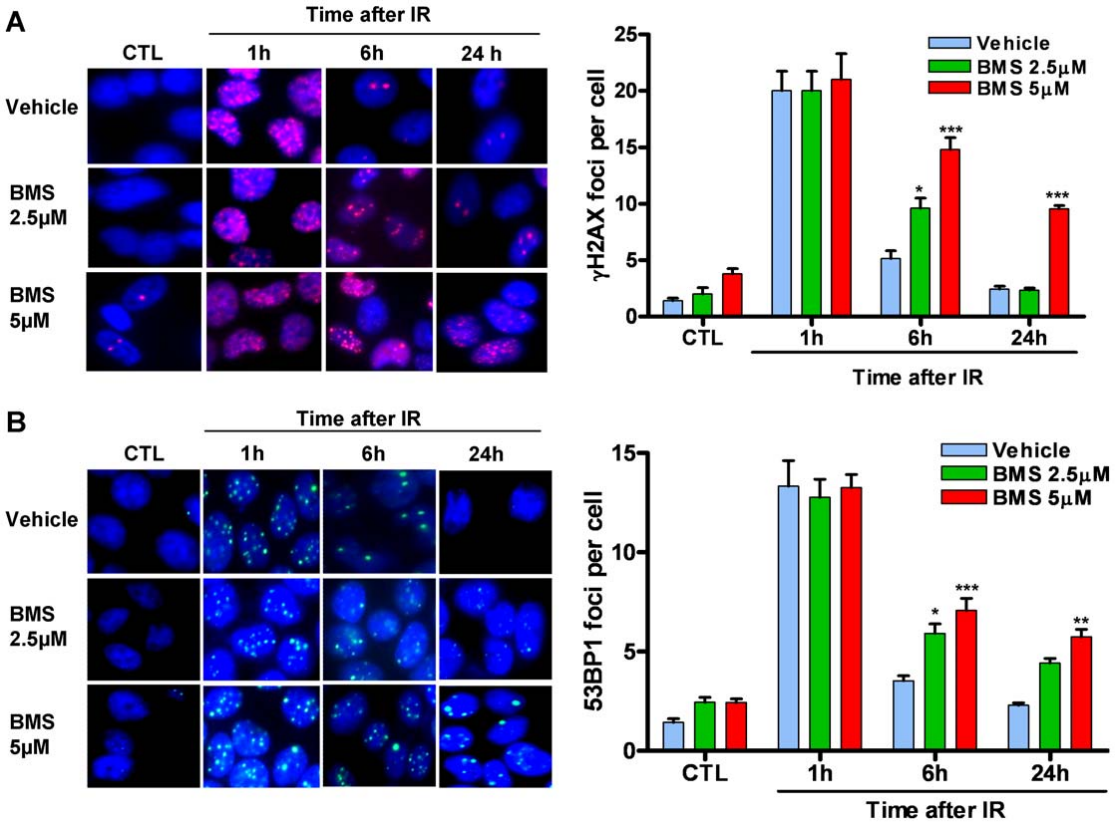
IKKβ inhibitor suppresses the repair of IR-induced DSBs. MCF-7 cells were incubated with vehicle (0.1% DMSO) or 2.5 and 5 µM BMS-345541 (BMS) for 1 h before exposure to 2 Gy IR. DSBs were analyzed by γH2AX and 53BP1 immunofluorescent staining at various time points after IR. *(A)* Representative photomicrographs (100× magnifications) of γH2AX immunofluorescent staining (red) and nucleic counterstaining with Hoechst-33342 (blue) are shown in the left panel and the average numbers of γH2AX foci/cell from three independent experiments are presented in the right panel. *(B)* Representative photomicrographs (100× magnifications) of 53BP1 immunofluorescent staining (green) and nucleic counterstaining with Hoechst-33342 (blue) are shown in the left panel and the average numbers of 53BP1 foci/cell from three independent experiments are presented in the right panel. The data are presented as mean ± SE. * p<0.05, ** p<0.01, and *** p<0.001, vs. vehicle.

**Figure 2:**
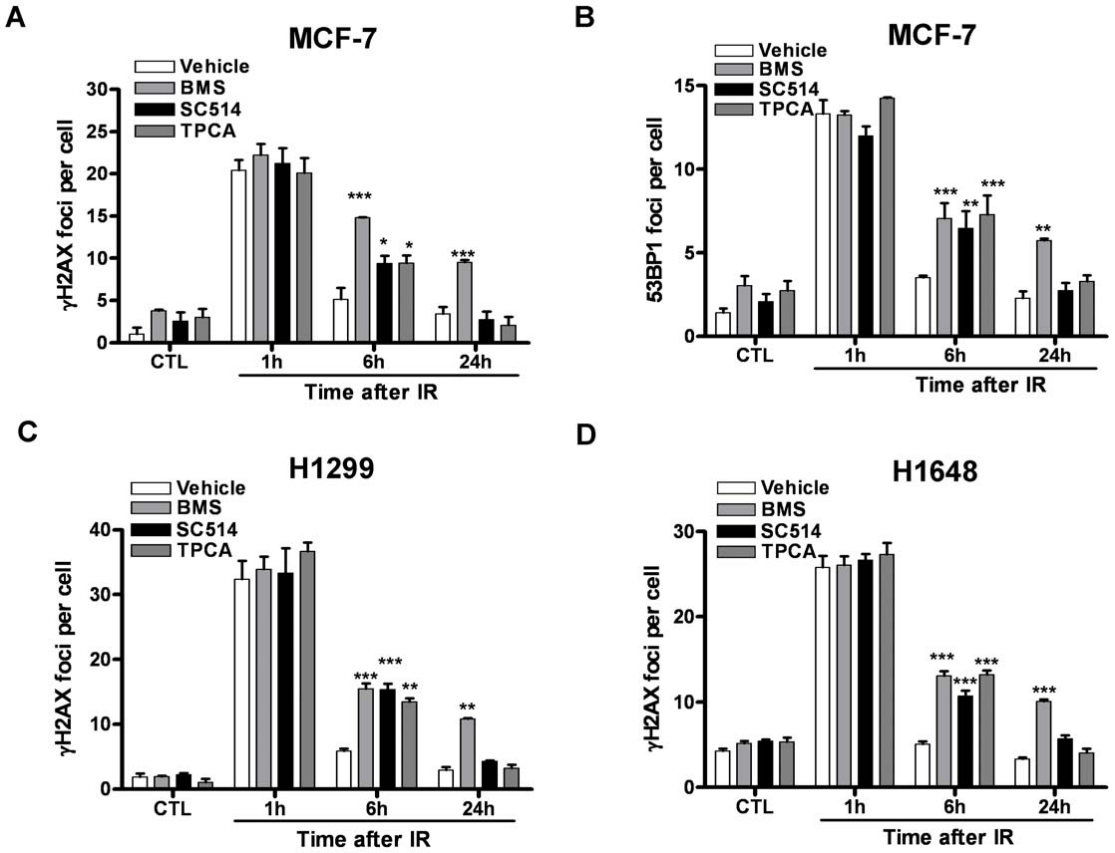
Effects of different IKKβ inhibitors on DSB repair in different cancer cell lines. *(A)* and *(B)* MCF-7 cells were incubated with vehicle (0.1% DMSO), 5 µM BMS-345541 (BMS), 5 µM TPCA-1 or 25 µM SC514 for 1 h before exposure to 2 Gy IR. DSBs were analyzed by γH2AX and 53BP1 immunofluorescent staining at various time points after IR. Un-irradiated cells were included as controls (CTL). The average numbers of γH2AX and 53BP1 foci/cell from three independent experiments are presented as mean ± SE. *(C)* and *(D)* H1299 and H1648 cells were incubated with vehicle (0.1% DMSO) or 5 µM BMS-345541 (BMS), 5 µM TPCA-1 or 25 µM SC514 for 1 h before exposure to 2 Gy IR. DSBs were analyzed by γH2AX immunofluorescent staining at various time points after IR. The average numbers of γH2AX foci/cell from three independent experiments are presented as mean ± SE. *p<0.05, **p<0.01, and *** p<0.001, vs. vehicle.

Activation of NFκB by IR depends on IKKβ [6]. BMS is a potent and specific IKKβ inhibitor and can effectively inhibit NFκB activation induced by diverse stimuli [10]. Therefore, we treated MCF-7 cells with BMS to determine whether activation of the IKKβ-NFκB pathway regulates the repair of IR-induced DSBs by γH2AX foci assay [11]. As shown in Figure 1 A, MCF-7 cells exhibited a low level of DSBs prior to exposure to IR. The basal levels of DSBs were not significantly changed after incubation with BMS (p>0.05). Exposure to IR increased DSBs in MCF-7 cells and the increases were comparable in the cells treated with vehicle or BMS 1 hr after IR when the formation of γH2AX foci reached the peak level (Figure 1 A). The numbers of γH2AX foci in vehicle-treated cells declined rapidly thereafter and were almost back to the basal level at 6 hr after IR, indicating that these cells can efficiently repair IR-induced DSBs. In contrast, the numbers of γH2AX foci in BMS-treated cells remained significantly elevated 6 hr after IR. Moreover, even up to 24 hr after IR MCF-7 cells treated with 5 µM BMS still exhibited a significant increase in γH2AX foci. Similar findings were also observed when the formation of 53BP1 foci was used as an alternative surrogate to quantify IR-induced DSBs, as 53BP1 can be rapidly recruited by γ H2AX to the sites of DSBs to form 53BP1 foci (Figure 1 B) [12]. These findings demonstrate that BMS can inhibit the repair of IR-induced DSBs in MCF-7 cells. To determine whether the effect of BMS is specific to MCF-7 cells and whether other IKKβ inhibitors have a similar effect as BMS, we extended the studies to two additional human lung cancer cell lines H1299 and H1648 and two other potent IKKβ inhibitors SC-514 [13] and TPCA-1 [14] and observed similar results as seen in MCF-7 cells treated with BMS (Figure 2). However, among these inhibitors examined, BMS is the most potent inhibitor of DSB repair.

### BMS is equally potent as DNA-dependent protein kinase (DNA-PK) and ATM inhibitors in inhibition of DSB repair

**Figure 3:**
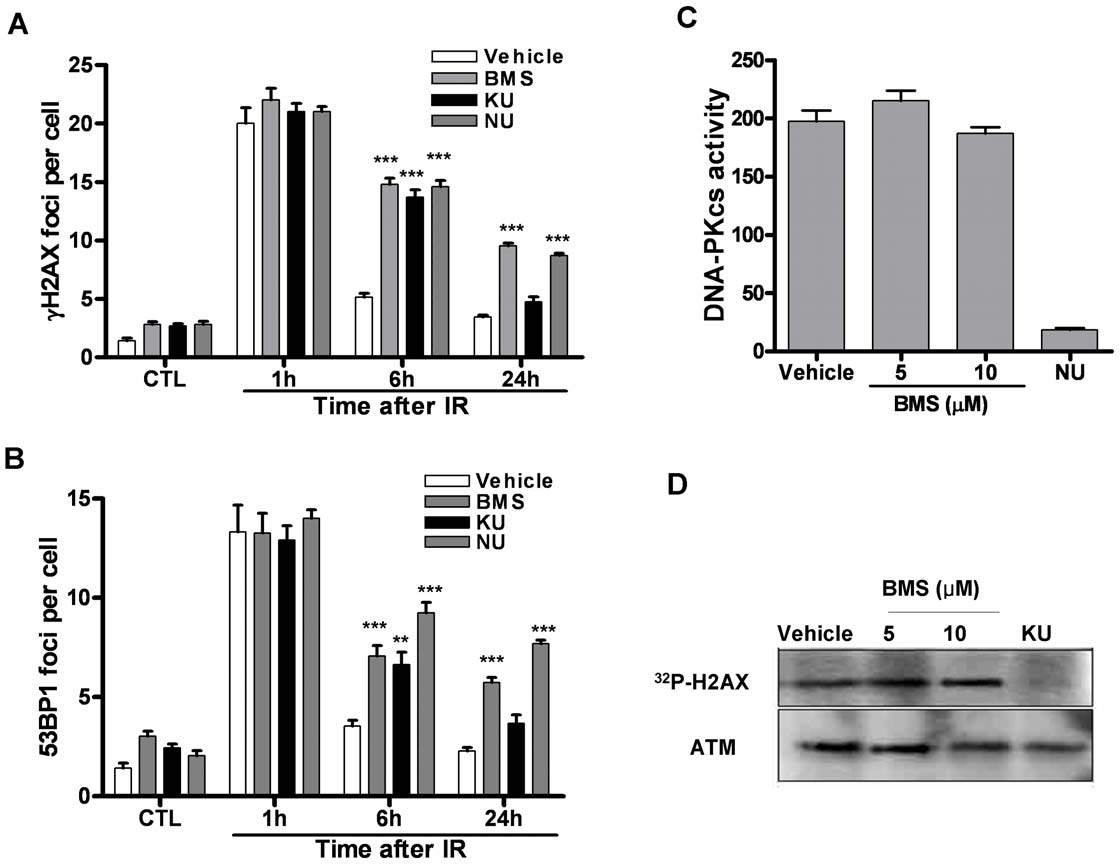
IKKβ inhibitor is equally potent as DNA-PK and ATM inhibitors in inhibition of the repair of IR-induced DSBs. *(A)* and *(B)* MCF-7 cells were incubated with vehicle (0.1% DMSO) or 5 µM BMS-345541 (BMS), NU-7026 (NU) or KU-55933 (KU) for 1 h before exposure to 2 Gy IR. DSBs were analyzed by γH2AX and 53BP1 immunofluorescent staining before IR (CTL) or at various time points after IR. The average numbers of γH2AX and 53BP1 foci/cell from three independent experiments are presented as mean ± SE. *** p<0.001, vs. vehicle. *(C)* DNA-PK kinase activity assay. The kinase activity was calculated according to the radioactivity of the γ-^32^P-substrate. The data are presented as mean ± SE (n = 3). *** p<0.001, vs. vehicle. *(D)* ATM kinase activity assay. A representative of γ-^32^P-H2AX autoradiography and ATM Western blot is shown. Similar results were observed in two additional experiments.

NU7026 (NU) and KU55933 (KU) are well characterized DNA-PK and ATM inhibitors, respectively [15], [16]. Both of them can potently inhibit DSB repair and sensitize various tumor cells to IR. Therefore, we compared the inhibitory effect of BMS with these of NU and KU on the repair of IR-induced DSBs. As shown in Figure 3 A and B, MCF-7 cells exhibited similar increases in γH2AX and 53BP1 foci 1 hr after IR in regardless of their pre-treatment. At 6 hr after IR, the majority of IR-induced DSBs were repaired in vehicle-treated MCF-7 cells, whereas significantly fewer DSBs were repaired in the cells treated with BMS, NU or KU. Even at 24 hr after IR, substantial DSBs remained unrepaired in MCF-7 cells treated with BMS and NU. These findings demonstrate that BMS is equally potent as DNA-PK and ATM inhibitors in inhibition of the repair of IR-induced DSBs.

Although BMS is a selective inhibitor of IKKβ, it is not know whether it inhibits DNA-PK and ATM [10]. Therefore, we examined the effects of BMS on DNA-PK and ATM in *in vitro* kinase assays. As shown in Figure 3 C and D, 5 µM NU and KU almost completely inhibited the kinase activities of DNA-PK and ATM, respectively. However, the same concentration of BMS had no such effect. Even at a higher concentration (10 µM), the kinase activities of DNA-PK and ATM remained unaffected by BMS. This result suggests that the inhibition of DSB repair by BMS is unlikely attributed to a non-specific inhibition of DNA-PK and ATM.

### IKKβ is essential for efficient repair of IR-induced DSBs

**Figure 4:**
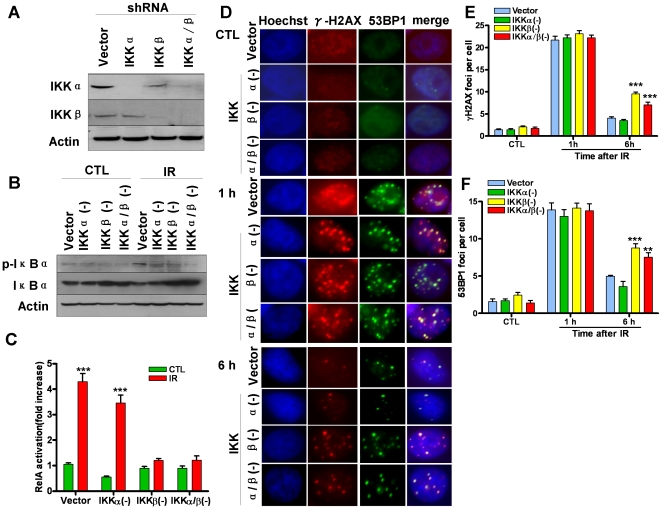
IKKβ is essential for the repair of IR-induced DSBs. *(A)* Down-regulation of IKKα and/or IKKβ expression in MCF-7 cells by shRNA was confirmed by Western blot analysis. *(B)* Down-regulation of IKKα and/or IKKβ expression by shRNA inhibits IR-induced phosphorylation of IκBα in MCF-7 cells. The levels of phosphorylated IκBα (p-IκBα) in the lysates from vector- or IKKα and/or IKKβ shRNA-transfected MCF7 cells before (CTL) or 30 min after IR (2 Gy) were analyzed by Western blots. *(C)* Down-regulation of IKKα and/or IKKβ expression by shRNA inhibits IR-induced NFκB activation in MCF-7 cells. NFκB activation was analyzed by quantification of the levels of RelA in the nuclear extracts from vector- or IKKα and/or IKKβ shRNA-transfected MCF7 cells before (CTL) or 30 min after IR (2 Gy) by an ELISA assay. The data are presented as mean ± SE (n = 3). *** p<0.001, vs. CTL. *(D)*-*(F)* Down-regulation of IKKβ but not IKKα expression by shRNA inhibits the repair of IR-induced DSBs in MCF-7 cells. DSBs were analyzed by γH2AX and 53BP1 immunofluorescent staining at 1 h and 6 h after vector- or IKKα and/or IKKβ shRNA-transfected MCF7 cells were exposed to 2 Gy IR. Un-irradiated cells were included as controls (CTL). Representative photomicrographs (100× magnifications) of γH2AX (red) and 53BP1 (green) immunofluorescent staining and nucleic counterstaining with Hoechst-33342 (blue) are shown in *(D)* and the average numbers of γH2AX and 53BP1 foci/cell from three independent experiments are presented *(E)* and *(F)* as mean ± SE. ** p<0.01, and *** p<0.001, vs. vector-transfected cells.

To further explore the requirement of IKK in efficient DSB repair, we generated stable IKKα and/or IKKβ knockdown cell lines, e.g. IKKα(-), IKKβ(-), and IKKα/β(-) cells, by transfection of MCF-7 cells with lentiviral short hairpin RNAs (shRNAs) that specifically target IKKα and/or IKKβ mRNA. As shown in Figure 4 A–C, down regulation of IKKα and/or IKKβ expression significantly inhibited IR-induced NFκB activation. Interestingly, IKKβ(-) and IKKα/β(-) cells, but not IKKα(-) cells, exhibited a significant reduction in the repair of IR-induced DSBs (Figure 4 D and F). These findings suggest that IKKβ, but not IKKα is essential for DSB repair, which was confirmed by the observations from IKKα and/or IKKβ knockout mouse embryonic fibroblasts (MEFs) as well (Figure S1).

**Figure 5:**
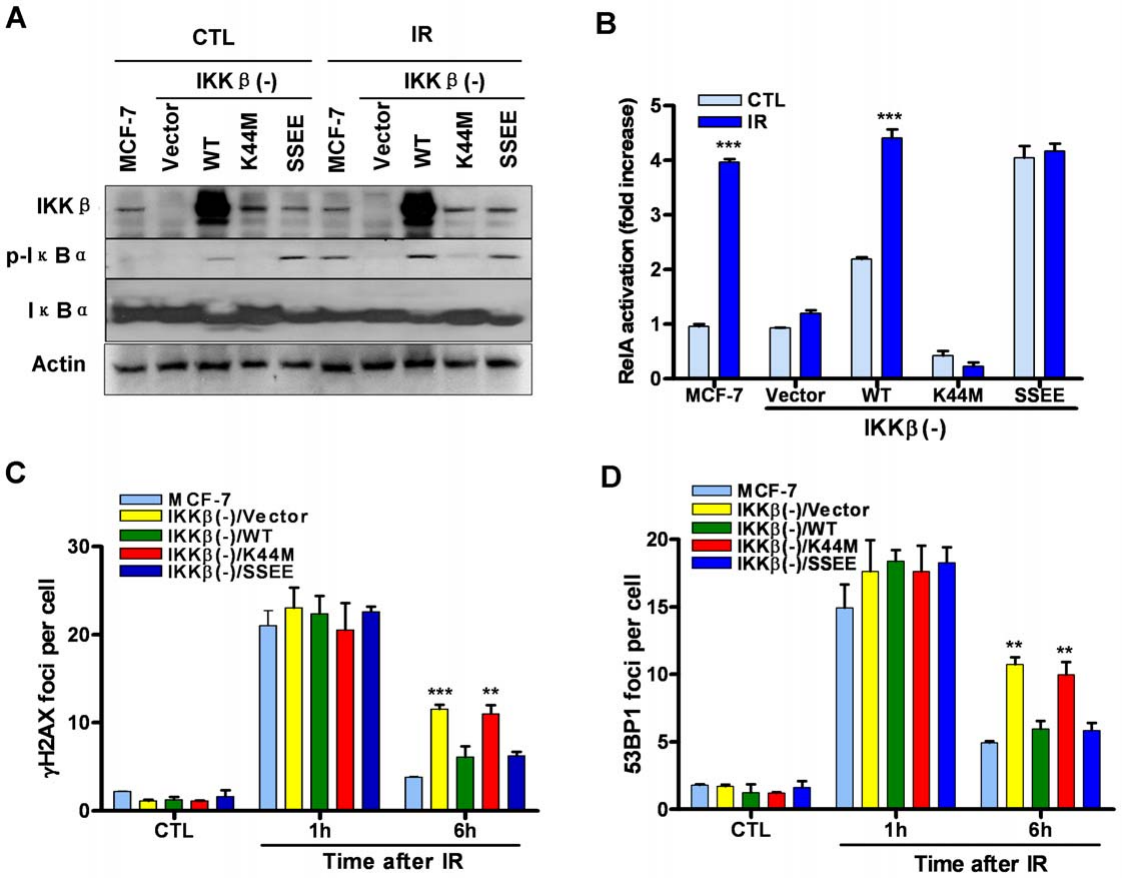
Reconstitution of IKKβ to MCF-7/IKKβ(-) cells restores DSB repair function. Analysis of IKKβ expression and IR-induced phosphorylation of IκBα *(A)* and NFκB activation *(B)* in MCF-7 cells and MCF-7/IKKβ (-) cells transfected with vector, wild-type (WT), kinase dead (K44M), or a constitutively active form (SSEE) IKKβ before (CTL) or 30 min after IR (2 Gy) by Western blots and an ELISA assay, respectively. The data presented in *(B)* are mean ± SE (n = 3). *** p<0.001 vs. CTL. *(C)* and *(D)* Reconstitution of IKKβ to MCF-7/IKKβ(-) cells restores DSB repair function. DSBs were analyzed by γH2AX and 53BP1 immunofluorescent staining at 1 h and 6 h after MCF-7 cells and MCF-7/IKKβ (-) cells transfected with vector, wild-type (WT), kinase dead (K44M), or a constitutively active form (SSEE) IKKβ were exposed to 2 Gy IR. Un-irradiated cells were included as controls (CTL). The average numbers of γH2AX and 53BP1 foci/cell from three independent experiments are presented in *(C)* and *(D)*, respectively, as mean ± SE. ** p<0.01, and *** p<0.001, vs. MCF-7 cells.

To further validate the essential role of IKKβ in DSB repair and determine whether the kinase activity of IKKβ is required for the regulation, we reconstituted MCF-7/IKKβ(-) cells with wild-type IKKβ (WT- IKKβ), kinase-dead IKKβ (K44M-IKKβ), and constitutively active IKKβ (SSEE-IKKβ) [17] by lentiviral transfection. The expression of these respective transgenes was confirmed by Western blot as shown in Figure 5 A. As reported in a previous study the cells transfected with WT-IKKβ exhibited constitutive activation of the NFκB pathway as those transfected with SSEE-IKKβ [17] and the activation could not be augmented by IR (Figure 5 A and B). In contrast, the cells transfected with K44M-IKKβ exhibited a similar deficiency in NFκB activation as vector-transfected MCF-7/IKKβ(-) cells. The DSB repair function was completely restored in MCF-7/IKKβ(-) after transfection with either WT-IKKβ or SSEE-IKKβ compared to MCF-7 cells, while MCF-7/IKKβ(-)/K44M-IKKβ cells remained deficient in the repair of IR-induced DSBs as vector-transfected MCF-7/IKKβ(-) cells (Figure 5 C and D). These results along with the data from BMS experiments confirmed that IKKβ is critical for DSB repair and its kinase activity is indispensable for this function.

### Inhibition of IKKβ sensitizes MCF-7 cells to IR

**Figure 6:**
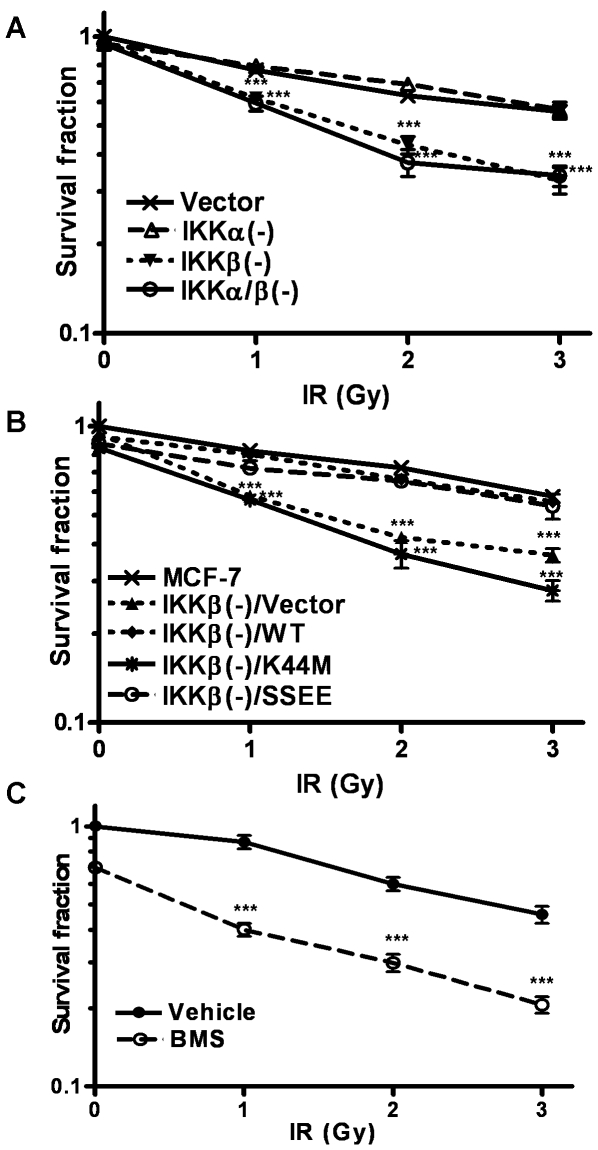
Inhibition of IKKβ sensitizes MCF-7 cells to IR. *(A)* Clonogenic survival of vector- or IKKα and/or IKKβ shRNA-transfected MCF7 cells after exposure to 0, 1, 2 and 3 Gy IR. *(B)* Clonogenic survival of MCF-7 cells and MCF-7/IKKβ (-) cells transfected with vector, wild-type (WT), kinase dead (K44M), or a constitutively active form (SSEE) IKKβ after exposure to 0, 1, 2 and 3 Gy IR. *(C)* Clonogenic survival of vehicle- or 2.5 µM BMS-345541 (BMS)-pretreated MCF-7 cells after exposure to 0, 1, 2 and 3 Gy IR. The data are expressed as mean ± SE (n = 3) of survival fraction compared to un-irradiated MCF-7 cells. *** p<0.001.

Since efficient repair of IR-induced DSBs is required for the clonogenic survival of irradiated cells [8], [9], we hypothesized that suppression of DSB repair via inhibition of IKKβ kinase activity can sensitize tumor cells to IR. To test this hypothesis, we exposed MCF-7, MCF-7/IKKα(-), MCF-7/IKKβ(-), and MCF-7/IKKα/β(-) cells to 0, 1, 2 and 3 Gy IR, which led to a dose-dependent reduction in their survival rate (Figure 6 A). The reduction was greater in MCF-7/IKKβ(-) and MCF-7/IKKα/β(-) cells than that in MCF-7 and MCF-7/IKKα(-) cells. Reconstitution of MCF-7/IKKβ(-) with WT-IKKβ or SSEE-IKKβ restored their resistance to IR, whereas MCF-7/IKKβ(-)/K44M-IKKβ cells remained equally sensitive to IR as vector-transfected MCF-7/IKKβ(-) cells (Figure 6 B). In addition, pharmacological inhibition of IKKβ kinase activity with BMS also sensitized MCF-7 cells to IR-induced clonogenic cell death (Figure 6 C). These findings suggest that inhibition of IKKβ activity sensitizes MCF-7 cells to IR at least in part via inhibition of DSB repair. Altogether our data support the notion that activation of IKKβ promotes the repair of DSBs and suppression of IKKβ activity inhibits the repair of IR-induced DSBs and sensitizes certain cancer cells to IR-induced cell death.

## Discussion

IR is one of the most widely used therapeutic modalities for cancer. Unfortunately, many tumor cells are inherently more resistant to IR or can acquire radioresistance shortly after radiotherapy, which inevitably leads to treatment failure and relapse of the disease [4]. An accumulating body of evidence suggests that constitutive activation of the IKK-NFκB pathway can contribute to cancer development, progression and resistance to cancer therapy [2], [3], whereas activation of this pathway by IR can also render tumor cells more resistant to radiotherapy [4]. Therefore, inhibition of the IKK-NFκB pathway has the potential to increase the therapeutic index of radiotherapy [4].

Among various inhibitors of the IKK-NFκB pathway, IKKβ inhibitors have emerged as the most promising anti-tumor agents and novel tumor sensitizers for IR and chemotherapy [13]. However, the mechanisms of their action have not been well studied but are presumably attributed to the inhibition of NFκB activity, which can increase tumor cell apoptosis by reducing the expression of anti-apoptotic proteins. The results from our studies reveal that IKKβ inhibitors can also inhibit the repair of IR-induced DSBs. This effect is not due to a non-specific inhibition of DNA-PK and ATM but specific inhibition of IKKβ, because DSB repair was also significantly inhibited by silencing IKKβ expression but not by IKKα knockdown and the repair function was restored after reconstitution of a functional IKKβ. Therefore, our results revealed a previously undescribed and important IKKβ kinase function, e.g. regulation of DSB repair.

It has been well established that IR kills cancer cells primarily by induction of DSBs. DSBs are considered the most detrimental DNA lesions and a single unrepaired DSB is sufficient to kill a cell. Therefore, targeted inhibition of the DSB repair pathways has been actively pursued as a way to sensitize tumor cells to IR and other chemotherapeutic agents [8], [9]. KU-55933 and NU-7026 are two well studied tumor sensitizers that inhibit DSB repair by targeting ATM and DNA-PK, respectively [15], [16]. We found that the potency of the IKKβ inhibitor BMS in inhibiting DSB repair is comparable to that of KU and NU. Since IKKβ inhibitors such as BMS can inhibit not only DSB repair but also NFκB-mediated induction of anti-apoptotic proteins [2], [3], [18], they are potentially more advantageous than ATM and DNA-PK inhibitors as a radiosensitizer. Interestingly, even though BMS is cytotoxic to some tumor cells and can sensitize MCF-7 human breast cancer cells to IR, it is a relatively safe agent that does not cause noticeable normal tissue damage *in vivo* [19]–[21]. These findings highlight the therapeutic potential of IKKβ inhibitors as an anti-tumor agent and a tumor sensitizer.

The mechanisms by which IKKβ regulates DSB repair have yet to be elucidated. Our preliminary data showed that selective inhibition of the NFκB transcriptional activity by ectopical expression of a mutant IκBα or down-regulation of RelA by RNAi had no effect on the repair of IR-induced DSBs (Figure S2), indicating that the induction of NFκB-RelA activity is not required for the regulation of DSB repair. However, it remains to be determined if activation of the other members of the NFκB family by IKKβ, such as c-Rel, may be involved in the regulation of DSB repair. For example, a recent report showed that activation of IKKβ up-regulates the expression of Claspin via c-Rel [22]. Claspin can regulate DNA damage-activated checkpoint response by promoting ataxia telangiectasia and Rad3-related protein (ATR)-mediated Chk1 phosphorylation and activation [23], [24]. However, it may not be unexpected to find that IKKβ may regulate DSB repair independent of NFκB, because several non-IκB targets of IKKβ have been identified recently [25], [26]. For example, it has been shown that IKKβ can directly phosphorylate Aurora kinase A to regulate its stability for the maintenance of bipolar sindle assembly and genomic stability [23]. In addition, a recent study showed that IKKβ translocates to the nucleus following UV irradiation [27]. It is plausible that IKKβ enters the nucleus following IR treatments to assist DSB repair processes. Alternatively, it will be interesting to determine if IKKβ-dependent DSB repair could be initiated by a mechanism involving the cytoplasmic IKKβ-ATM axis [6], [7], [28]. Identification of IKKβ substrate(s) required for DSB repair and elucidation of the mechanisms by which IKKβ regulates DSB repair will therefore opens up a new model of DNA damage response in mammalian cells, which will be investigated in our future studies.

In conclusion, for the first time, we demonstrate that IKKβ regulates the repair of IR-induced DSBs. Moreover, IKKβ, but not IKKα, is primarily responsible for promoting survival of certain tumor cells after IR at least in part by facilitating DSB repair. Surprisingly, NFκB-RelA is dispensable for IKKβ-dependent repair of DSBs. Therefore, IKKβ inhibition or critical processes involved in the IKKβ-dependent DSB repair pathway may be exploited as a novel therapeutic strategy to increase the sensitivity of tumor cells to IR.

## Materials and Methods

### Reagents

TPCA-1, SC-514 and NU-7026 (NU) were purchased from Calbiochem (San Diego, CA). KU-55933 (KU), BMS-345541 (BMS), Hoechst-33342 (Hoe) and Propidium iodide (PI) were purchased from Sigma-Aldrich (St. Louis, MO).

### Cell lines

MCF-7, H1299 and H1648 cell lines were originally obtained from ATCC (Manassas, VA). They were selected for our study because IR is a common therapeutic modality for breast and lung cancer and these cell lines have been extensively used in radiation research. MCF-7 cells stably transfected with the dominant-negative mutant IκBα (mIκBα or IκBα A32/36) were kindly provided by Dr. Jian Jian Li (University of California at Davis, Sacramento, CA). Immortalized wide type, IKKα, IKKβ, and IKKα/β double knockout mouse embryonic fibroblasts were kindly provided by Dr. Shigeki Miyamoto (University of Wisconsin-Madison, Madison, WI) with the permission of Dr. Inder Verma (Salk Institute, La Jolla, CA). All these cells were maintained in Dulbecco's modified Eagle's minimum (DMEM) supplemented with 10% fetal bovine serum (HyClone, Logan, UT), penicillin (100 U/ml) and streptomycin (100 µg/ml) in a humidified incubator (95% air/5% CO_2_) at 37°C.

### Construction of various IKKβ expression vectors

PLVUT-tTR-KRAB lentiviral vector (Addgene, Cambridge, MA) was digested with EcoRI and then ligated with synthetic oligonucleotides containing PmeI, HpaI and BstBI sites to generate PLVUT-1 vector. The PCR fragment containing PCMV IE-GFP and BstBI sites was generated from pEGFP-C1 vector (Clontech, Mountain View, CA) by PCR using Phusion® Hot Start High-Fidelity DNA Polymerase (New England Biolabs, Ipswich, MA). It was inserted into BstBI site of PLVUT-1 vector to generate PLVUT-GFP vector. According to the sequences of IKKβ/WT, IKKβ/K44M and IKKβ/SSEE vectors (Addgene), IKKβ/WT, IKKβ/K44M and IKKβ/SSEE fragments containing PmeI and HpaI sites were generated by PCR using Phusion® Hot Start High-Fidelity DNA Polymerase. PLVUT-GFP-IKKβ/WT, PLVUT-GFP-IKKβ/K44M, PLVUT-GFP-IKKβ/SSEE lentiviral vectors were constructed after insertion of these fragments into PmeI and HpaI sites of PLVUT-GFP vector. The resulting vectors were confirmed by DNA sequence.

### Lentivirus production

Lentivirus was produced after transient infection of human embryonic kidney (HEK) 293T cells with individual lentiviral vectors along with the packaging plasmids pCMV-VSV-G and psPAX2 (Addgene) using FuGEN6-HD (Roche Diagnostics, Mannheim, Germany) as the infection reagent according to Roche's protocol. The supernatants containing viral particles were collected 48 h after the infection and filtered through 0.22 µm filter. The viral particles were concentrated using a kit (PEG-itTM Virus Precipitation Solution) from System Biosciences (Mountain View, CA) according to the manufacturer's instructions.

### Ionizing radiation

Cells were exposed to various doses of IR in a JL Shepherd Model 143 ^137^Cesium -irradiator (JL Shepherd, Glendale, CA) at a dose rate of 2.4 Gy/min. Cells were irradiated on a rotating platform.

### Immunofluorescence staining

Cells grown on a 4-chamber CultureSlide (BD Falcon, Bedford, MA) after various treatments were fixed, permeabilized, and stained as previously described [29]. The following antibodies were used for the staining: 1∶1000 mouse anti-phospho-H2AX (γH2AX [Ser139], clone JBW301; Millipore, Billerica, MA) or rabbit anti-53BP1 (cat# ab36823, from Abcam Inc., Cambridge, CA) and 1∶500 Alexa Fluor 568-conjugated anti-mouse IgG (Cat#A11004, from Invitrogen, Camarillo, CA) or FITC-conjugated anti-rabbit IgG (Cat# ab6767, from Abcam). Nuclei were counterstained with Hoechst-33342. Approximately 200 nuclei images were acquired using a Zeiss Axio Observer.Z1 microscope with an Apo 60X/1.4 oil DICIII objective and AxioVision (4.7.1.0) software (Carl Zeiss Microimaging Inc., GmbH, Jena; Germany). The numbers of γH2AX and/or 53BP1 foci for each cell was accounted, averaged and expressed as γH2AX and/or 53BP1 foci/cell.

### DNA-PK kinase assay

The kinase activity of DNA-PK was measured using a SignaTECT DNA-Dependent Protein Kinase Assay System (Cat# V7870, Promega, Madison, WI). The biotinylated peptide substrate was incubated with 50 units purified DNA-PK (Cat# V5811, Promega) and (γ-^32^P)ATP in the presence or absence of BMS (5 or 10 µM) or NU (5 µM) for 5 min at 30°C according to the manufacturer's instructions. The biotinylated substrate was captured on a streptavidin membrane, washed and quantified by a Storm 860 Phosphorimager (Molecular Dynamics, Sunnyvale, CA).

### ATM kinase assay

ATM was purified from irradiated MCF-7 cells by immunoprecipitation with anti-ATM antibody (Cat#A300-135A, from BETHYL Laboratories, Montgomery, TX) as previously described [30]. Aliquots of the purified ATM were incubated with 500 ng of recombinant H2AX (kindly provided by Dr. Benjamin Chen, University of Texas Southwestern Medical Center, Dallas, TX), 2 µl of 100 µM ATP and 10 µCi of (γ-^32^P)ATP in the presence or absence of BMS (5 or 10 µM) or KU (5 µM) at 30°C for 10 min. After SDS-polyacrylamide gel electrophoresis, γ-^32^P-H2AX was visualized by autoradiography.

### Knockdown of IKKα and/or IKKβ with short hairpin RNA (shRNA)

Control lentiviral pLKO.1 vector and pLKO.1 vectors containing shRNAs for human IKKα (RHS4533-NM_001278) and IKKβ (RHS4533-NM_001556) were obtained from Open Biosystems (Huntsville, AL). Viral particles were produced as described above. To establish stable IKKα and/or IKKβ knockdown MCF-7 cell lines, MCF-7 cells were infected twice with the viral particles under centrifugation (900×*g*) at 35°C for 30 min. Stably transduced cells were selected with puromycin (2 µg/ml). IKKα and/or IKKβ knockdown in MCF-7/IKKα(-), MCF-7/IKKβ(-) and MCF-7/IKKα/β(-) cells, respectively, was confirmed by Western blot.

### Reconstitution of IKKβ in MCF-7/IKKβ(-) cell lines

MCF-7/IKKβ(-) cells were infected twice with the viral particles containing PLVUT-GFP vector or the vector encoding IKKβ/WT, IKKβ/K44M and IKKβ/SSEE as described above. The cells expressing a moderate level of GFP were enriched twice by cell sorting to generate stable MCF-7/IKKβ(-)/vector, MCF-7/IKKβ(-)/WT, MCF-7/IKKβ(-)/K44M, and MCF-7/IKKβ(-)/SSEE cell lines. The expression of IKKβ in these cell lines was confirmed using Western blot.

### Western blot analysis

Total cell lysates were separated on 10% SDS–PAGE. The proteins were transferred onto a PVDF membrane, blocked with 5% nonfat milk for 1 h at room temperature, and probed with a primary antibody (1∶1000) overnight at 4°C. The primary antibodies used include anti-IKKα (Cat#2682), IKKβ (Cat# 2678) and RelA (Cat# 4764) from Cell Signaling Technology (Danvers, MA); and anti-phosphorylated IκBα (Cat# 2859), anti-IκBα (Cat# 4814) and Actin (Cat# SC-1616) from Santa Cruz Biotechnology (Santa Cruz, CA). The membranes were then incubated with a horseradish peroxidase-conjugated secondary antibody at 1∶10,000 dilutions (Jackson ImmunoResearch Labs., West Grove, PA) for 1 h. Protein bands were visualized using Amersham ECL Western Blotting Detection Reagents (GE Healthcare, Piscataway, NJ) and exposed to ECL Plus film (GE Healthcare).

### NFκB RelA DNA-binding activity assay

Nuclear proteins were extracted from cells using the Nuclear Extract Kit (from Active Motif, Carlsbad, CA) per the manufacturer's protocol and were not contaminated by cytoplasmic elongation factor 2 (EF2) based on the result of Western blot using an antibody against EF2. NFκB-RelA DNA-binding activity was determined by a TransAM^TM^ NFκB-RelA kit (Active Motif) using 5 µg of nuclear extract proteins according to the manufacturer's instructions.

### Down-regulation of RelA with small interference RNA (siRNA)

To down-regulate the expression of RelA with siRNA, 5×10^4^ MCF-7 cells were seeded into a well of six-well plates. After overnight incubation, the medium was removed and then replaced with transfection media containing control (siGENOME Non-Targeting siRNA, D-001210-02-05) or RelA (siGENOME SMARTpool siRNA, M-003533-02-0005) siRNA (final concentration 50 nmol/L) along with DharmaFECT4 transfection reagent (Dharmacon) according to the manufacturer's protocol. After 24 h incubation, the transfection medium was removed and replaced with cell culture medium. The cells were allowed to grow for an additional 48 h to achieve maximal knockdown of RelA as shown by real-time RT-PCR and Western blot analyses.

### Real-time PCR

Real-time PCR was done as previously described using the following primers: RelA, forward 5′-CCTTCCTCATCCCATCTT TG- 3′ and reverse 5′-CCTCAATGTCCTCTTTCTGC-3′; and GAPDH, forward 5′-CCC CAC ACA CAT GCA CTT ACC-3′ and reverse 5′-CCT ACT CCC AGG GCT TTG ATT-3′. The threshold cycle (Ct) value for RelA was normalized to the Ct value of GAPDH. The relative RelA mRNA expression was calculated using the comparative C_T_ (2^-ΔΔCt^) method as previously described [29].

### Clonogenic survival assay

MCF-7 cells were seeded into wells of 12-well plates at 1×10^4^ cells/well. After overnight incubation, they were exposed to various doses (0, 1, and 3 Gy) of IR with or without pretreatment as indicated in individual experiments. The cells were allowed to grow for additional 12 days to form colonies before stained with 0.1% crystal purple. Colonies with more than 50 cells were counted. Survival fraction was calculated according to the plating efficiency of control cultures.

### Statistical analysis

The data were analyzed by analysis of variance (ANOVA). In the event that ANOVA justified post hoc comparisons between group means, these were conducted using the Student-Newman-Keuls test for multiple comparisons. For experiments in which only single experimental and control groups were used, group differences were examined by unpaired Student *t* test. Differences were considered significant at *P<0.*05. All of these analyses were done using GraphPad Prism from GraphPad Software (San Diego, CA).

## Supporting Information

Figure S1 **IKKβ but not IKKα knockout inhibits the repair of IR-induced DSBs in mouse embryonic fibroblasts.** Mouse embryonic fibroblasts (MEF) from wild-type (WT), IKKα, IKKβ, and IKKα/β knockout mice were exposed to 2 Gy IR. DSBs were analyzed by γH2AX and 53BP1 immunofluorescent staining at 1 h and 6 h after IR. Un-irradiated cells were included as controls (CTL). Representative photomicrographs (100× magnifications) of γH2AX (red) and 53BP1 (green) immunofluorescent staining and nucleic counterstaining with Hoechst-33342 (blue) are shown in *(A)* and the average numbers of γH2AX and 53BP1 foci/cell from three independent experiments are presented *(B)* and *(C)* as mean ± SE. * p<0.05, ** p<0.01, and *** p<0.001, vs. WT MEFs.
(TIF)

Figure S2 **IKKβ regulates DSB repair in a NFκB-RelA independent manner.** *(A)* and *(B)* Ectopic expression of mIκBα inhibits IR-induced phosphorylation of IκBα and NFκB activation in MCF-7 cells. The levels of phosphorylated IκBα (p-IκBα) and total IκBα in the lysates from vector- or mIκBα-transfected MCF7 cells before (CTL) or 30 min after IR (2 Gy) were analyzed by Western blots. NFκB activation was analyzed by quantification of the levels of RelA in the nuclear extracts from vector- or mIκBα-transfected MCF7 cells before (CTL) or 30 min after IR (2 Gy) by an ELISA assay. The data presented in *(B)* are mean ± SE (n = 3). *** p<0.001, vs. vehicle. *(C)* Ectopic expression of mIκBα has no effect on the repair of IR-induced DSBs in MCF-7 cells. DSBs were analyzed by γH2AX immunofluorescent staining at 1 and 6 h after vector- or mIκBα-transfected MCF7 cells were exposed to 2 Gy IR. Un-irradiated cells were included as a control (CTL). The average numbers of γH2AX foci/cell from three independent experiments are presented as mean ± SE. *(D)* Down-regulation of RelA mRNA expression by siRNA was confirmed by real-time PCR. The expression of RelA and GAPDH mRNA in RelA siRNA-treated cells was expressed as a percentage of that in control siRNA-treated cells. The data are presented as mean ± SE (n = 3). *** p<0.001, vs. control siRNA treatment. *(E)* Down-regulation of RelA expression by siRNA was confirmed by Western blot in MCF-7 cells transfected with control (CTL) or RelA siRNA. Un-transfected MCF-7 cells (Control) were included as a control. *(F)* Down-regulation of RelA expression by siRNA has no effect on the repair of IR-induced DSBs in MCF-7 cells. DSBs were analyzed by γH2AX immunofluorescent staining at 1 and 6 h after control (CTL siRNA) or RelA siRNA-transfected MCF7 cells were exposed to 2 Gy IR. Un-irradiated cells were included as controls (CTL). The average numbers of γH2AX foci/cell from three independent experiments are presented as mean ± SE.
(TIF)
