# Supplementary figures and images for: IKKβ Regulates the Repair of DNA Double-Strand Breaks Induced by Ionizing Radiation in MCF-7 Breast Cancer Cells
Source: PLoS One. 2011 Apr 7;6(4):e18447. doi: 10.1371/journal.pone.0018447 (PMC3072401; doi:10.1371/journal.pone.0018447)

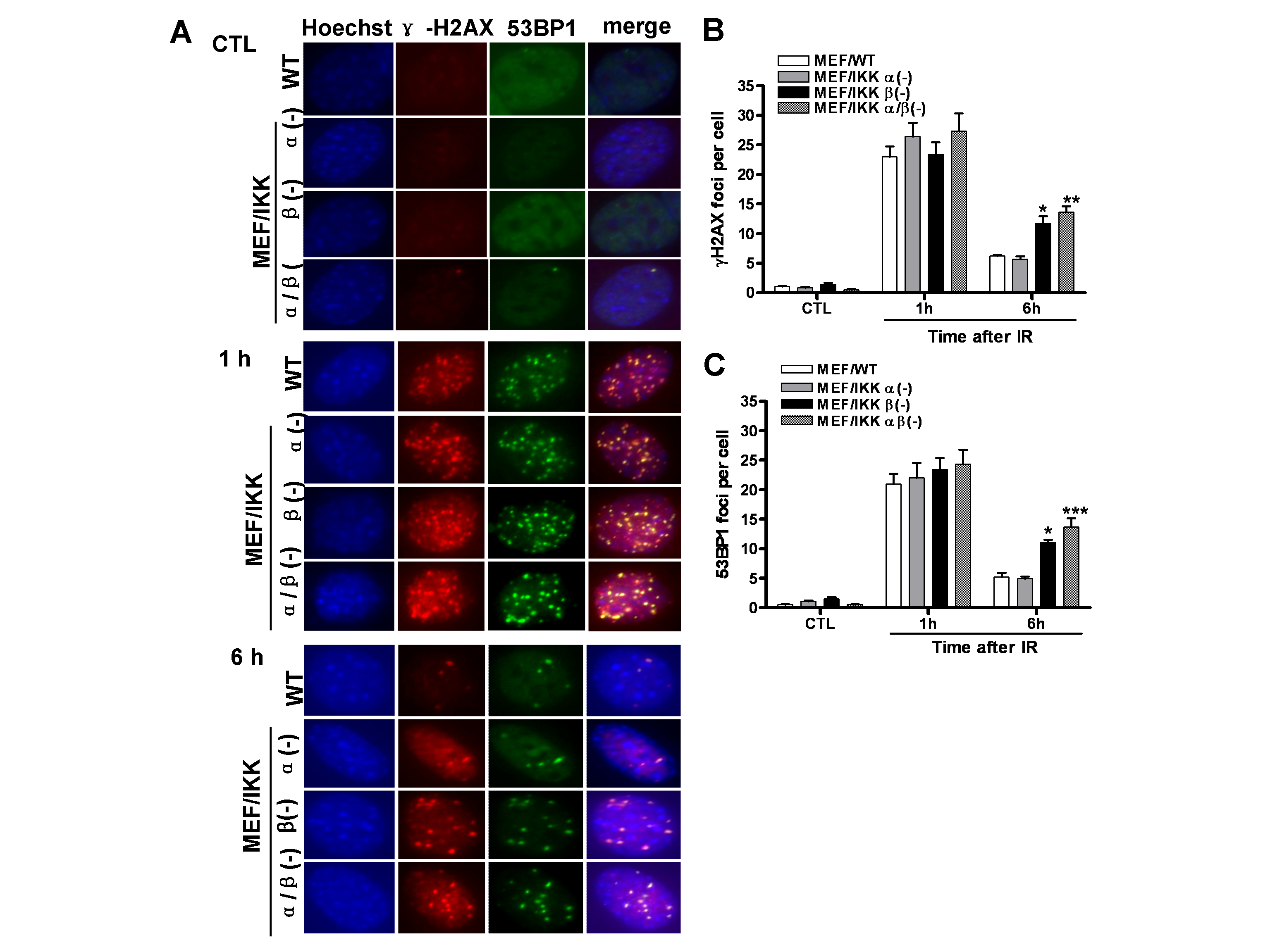

Supplement: Figure S1 — IKKβ but not IKKα knockout inhibits the repair of IR-induced DSBs in mouse embryonic fibroblasts. Mouse embryonic fibroblasts (MEF) from wild-type (WT), IKKα, IKKβ, and IKKα/β knockout mice were exposed to 2 Gy IR. DSBs were analyzed by γH2AX and 53BP1 immunofluorescent staining at 1 h and 6 h after IR. Un-irradiated cells were included as controls (CTL). Representative photomicrographs (100× magnifications) of γH2AX (red) and 53BP1 (green) immunofluorescent staining and nucleic counterstaining with Hoechst-33342 (blue) are shown in (A) and the average numbers of γH2AX and 53BP1 foci/cell from three independent experiments are presented (B) and (C) as mean ± SE. * p<0.05, ** p<0.01, and *** p<0.001, vs. WT MEFs. (TIF) [file pone.0018447.s001.tif]

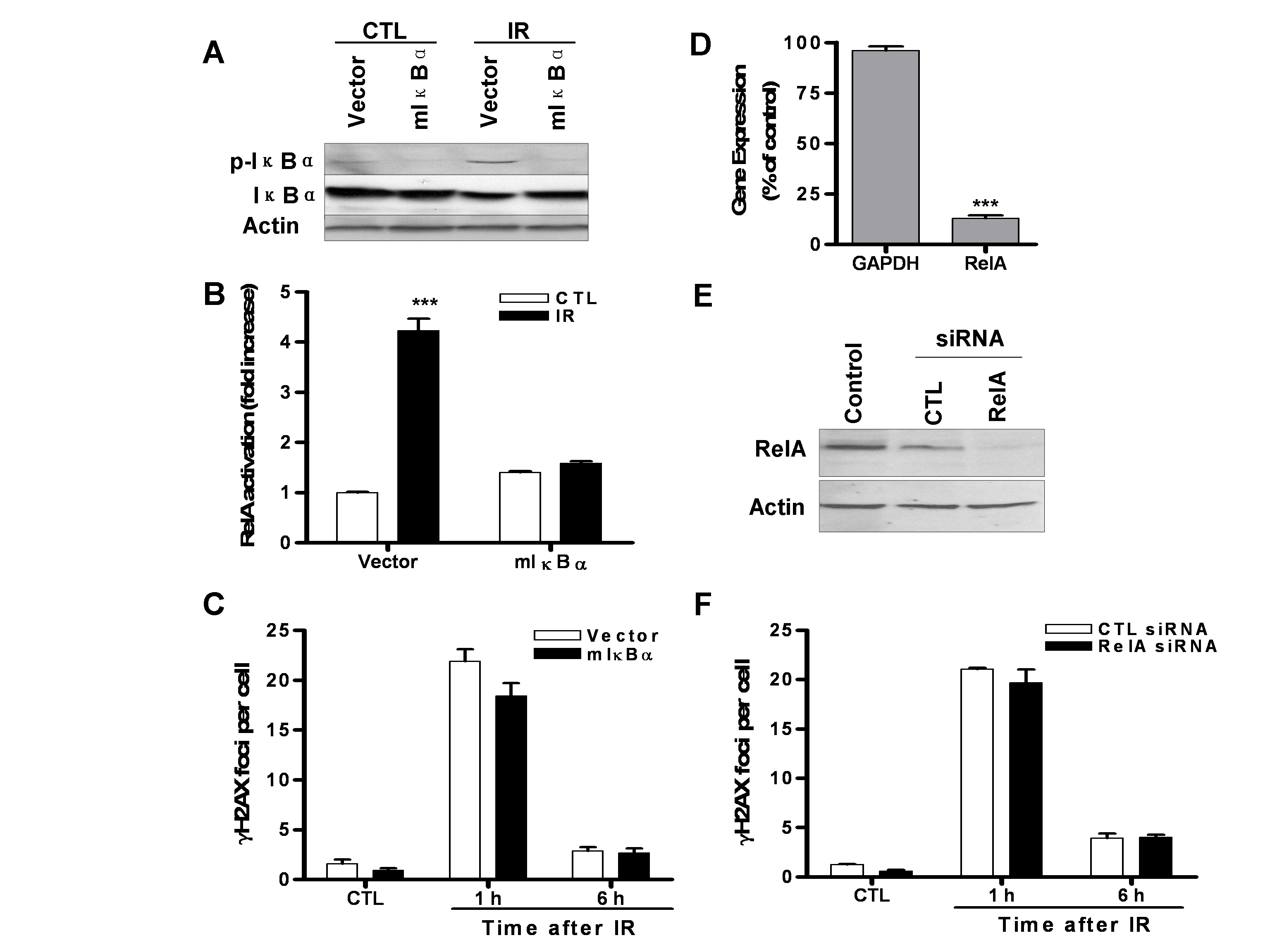

Supplement: Figure S2 — IKKβ regulates DSB repair in a NFκB-RelA independent manner. (A) and (B) Ectopic expression of mIκBα inhibits IR-induced phosphorylation of IκBα and NFκB activation in MCF-7 cells. The levels of phosphorylated IκBα (p-IκBα) and total IκBα in the lysates from vector- or mIκBα-transfected MCF7 cells before (CTL) or 30 min after IR (2 Gy) were analyzed by Western blots. NFκB activation was analyzed by quantification of the levels of RelA in the nuclear extracts from vector- or mIκBα-transfected MCF7 cells before (CTL) or 30 min after IR (2 Gy) by an ELISA assay. The data presented in (B) are mean ± SE (n = 3). *** p<0.001, vs. vehicle. (C) Ectopic expression of mIκBα has no effect on the repair of IR-induced DSBs in MCF-7 cells. DSBs were analyzed by γH2AX immunofluorescent staining at 1 and 6 h after vector- or mIκBα-transfected MCF7 cells were exposed to 2 Gy IR. Un-irradiated cells were included as a control (CTL). The average numbers of γH2AX foci/cell from three independent experiments are presented as mean ± SE. (D) Down-regulation of RelA mRNA expression by siRNA was confirmed by real-time PCR. The expression of RelA and GAPDH mRNA in RelA siRNA-treated cells was expressed as a percentage of that in control siRNA-treated cells. The data are presented as mean ± SE (n = 3). *** p<0.001, vs. control siRNA treatment. (E) Down-regulation of RelA expression by siRNA was confirmed by Western blot in MCF-7 cells transfected with control (CTL) or RelA siRNA. Un-transfected MCF-7 cells (Control) were included as a control. (F) Down-regulation of RelA expression by siRNA has no effect on the repair of IR-induced DSBs in MCF-7 cells. DSBs were analyzed by γH2AX immunofluorescent staining at 1 and 6 h after control (CTL siRNA) or RelA siRNA-transfected MCF7 cells were exposed to 2 Gy IR. Un-irradiated cells were included as controls (CTL). The average numbers of γH2AX foci/cell from three independent experiments are presented as mean ± SE. (TIF) [file pone.0018447.s002.tif]
